# Overview on the Antihypertensive and Anti-Obesity Effects of Secondary Metabolites from Seaweeds

**DOI:** 10.3390/md16070237

**Published:** 2018-07-14

**Authors:** Ana M. L. Seca, Diana C. G. A. Pinto

**Affiliations:** 1 cE3c—Centre for Ecology, Evolution and Environmental Changes/Azorean Biodiversity Group & Faculty of Sciences and Technology, University of Azores, Rua Mãe de Deus, 9501-321 Ponta Delgada, Portugal; 2 Department of Chemistry & QOPNA-Organic Chemistry, Natural Products and Food Stuffs, University of Aveiro, Campus de Santiago, 3810-193 Aveiro, Portugal; diana@ua.pt

**Keywords:** seaweeds, anti-hypertension, anti-obesity, peptides, phlorotannins, fucoxanthin

## Abstract

Hypertension and obesity are two significant factors that contribute to the onset and exacerbation of a cascade of mechanisms including activation of the sympathetic and renin-angiotensin systems, oxidative stress, release of inflammatory mediators, increase of adipogenesis and thus promotion of systemic dysfunction that leads to clinical manifestations of cardiovascular diseases. Seaweeds, in addition to their use as food, are now unanimously acknowledged as an invaluable source of new natural products that may hold noteworthy leads for future drug discovery and development, including in the prevention and/or treatment of the cardiovascular risk factors. Several compounds including peptides, phlorotannins, polysaccharides, carotenoids, and sterols, isolated from brown, red and green macroalgae exhibit significant anti-hypertensive and anti-obesity properties. This review will provide a comprehensive overview of the recent advances on bioactive pure compounds isolated from different seaweed sources focusing on their potential use as drugs to treat or prevent hypertension and obesity. On the other hand, although it is obvious that macroalgae represent promising sources of antihypertensive and anti-obesity compounds, it is also clear that further efforts are required to fully understand their cellular mechanisms of action, to establish structure-inhibition relationships and mainly to evaluate them in pre-clinical and clinical trials.

## 1. Introduction

Hypertension and obesity are key adverse health metrics that have disastrous health implications. Obesity, defined as excess body fat, is considered the gateway condition for several chronic diseases and is a major factor in the development of cardiovascular and metabolic disorders such as hypertension, ischaemic stroke, insulin resistance, impaired glucose tolerance, hyperinsulinemia and dyslipidaemia [1]. Hypertension, a high blood pressure condition called the “silent killer” as it can be asymptomatic for years before being clinically diagnosed, is a major modifiable risk factor of cardiovascular disease such as ischemic and haemorrhagic stroke, myocardial infarction, heart failure, chronic kidney disease, peripheral vascular disease, cognitive decline and premature death [2]. Although there are many unanswered questions about the causes of obesity and hypertension, it seems that they have common pathophysiological mechanisms.

Obesity augments sympathetic nerve traffic due to hyperinsulinemia and renal norepinephrine spillover, which increase renal tubular reabsorption of sodium and as a consequence active the renin-angiotensin system (RAS) [3,4]. Besides that, in the obesity process there are increase endothelial dysfunction and vascular oxidative stress attributed in part to circulating adipokines (increased production of leptin, decreased production of adiponectin), amplified reactive oxygen species, and reduced nitric oxide availability which together with endothelin and RAS are the most important factors regulating vascular tone [3,4,5]. The pathogenesis of hypertension is multifactorial and complex, being related to differing concentrations of sodium and potassium in the body, obesity, insulin resistance, high alcohol intake, low calcium intake, stress and ageing diseases. The three main factors that determine blood pressure are renal sodium excretion (and the resultant impact on plasma and total body volume), vascular tone and cardiac performance and these factors control the cardiac output, the intravascular volume and the systemic vascular resistance [3,6].

As a result of the mentioned pathophysiological mechanisms, obesity and hypertension are associated during their progression with the development of organ damage and consequent renal diseases (e.g., glomerulosclerosis, renal insufficiency), cardiac diseases (e.g., eccentric cardiac hypertrophy, heart failure), retinopathy and mainly vascular alterations (arterial stiffness and a reduction in arterial compliance and distensibility and small resistance arteries) which increase the risk of cerebrovascular and coronary heart diseases [2,3,4]. Additionally, recent evidence suggests there are sex differences in mechanisms of obesity and hypertension [7]. Currently, the main targets for the treatment of hypertension are calcium channel blockers, diuretics, and renin-angiotensin system (RAS) inhibitors [6,8,9]. In this last target, renin is the enzyme that converts the inactive angiotensinogen to the active angiotensin I. This vasodilator is converted, by the angiotensin-I converting enzyme (ACE I), to angiotensin II, a potent vasoconstrictor [6]. Thus, there are two main ways to control blood pressure: the direct inhibition of angiotensin I generation or the blockage of its conversion into angiotensin II. Therefore, renin and/or ACE I inhibition are considered the main targets for hypertension treatment and many research works have been published about natural and synthetic compounds inhibiting RAS [6,8,9,10].

Seaweeds, also known as macroalgae, are traditionally used as food, mainly in China, Japan and the Republic of Korea [11], and they have recently become a popular ingredient in some Western diets. Some facts contribute to their success as food, for example the association between seaweed dietary intake and longer life expectancy or lower incidence of certain diseases, such as cardiovascular diseases [12]. Additionally, in recent years, industries from different branches (textile, fuel, plastics, paint, varnish, cosmetics, pharmaceutical and food) have increased the attention devoted to macroalgae products such as secondary metabolites because seaweeds are bio-renewable, with a high rate of biomass production that does not compete with food crops, and with constituents that have great versatility of applications [13,14]. Among many other possible examples, the use of seaweed and some of its components as bio-resources for fuel production [15,16], the use of seaweed cellulose to produce new cellulose derivative fibbers [17], and the use of seaweed-derived polysaccharide-based composites for food packaging and pharmaceutical applications including tissue engineering, drug delivery, and wound dressing [18] can be emphasized. In fact, the biodiversity within red (Rhodophyta), green (Chlorophyta) and brown (Phaeophyta) macroalgae offers the possibility of finding a wide variety of compounds, like carbohydrates, protein and minerals, as well as a rich source of health-promoting secondary metabolites with interesting properties and applications [13,19,20,21], including prevention and treatment of cardiovascular diseases risk factors [22,23,24,25]. Most macroalgae products described in the scientific literature as having antihypertensive and/or anti-obesity effects are the whole extract (aqueous or alcoholic), or fractions rich in a particular type of compound (e.g., fucoidans, alginates, phlorotannins) [22,23,26,27]. Scientific studies on the efficacy of these products are quite advanced, including animal studies, human studies and some in-clinical studies. Although they are interesting works, they are not the subject of this review, so we draw our readers’ attention to excellent reviews on this subject [22,23,24,25,27,28]. These extracts/fractions’ chemical composition is not unequivocally known. This entails problems such as adulteration, product quality control, dose/effect variability, complex mechanism of action, and determination of the active ingredient, which are only overcome through the standardization and strict quality control of the product to be marketed.

On the other hand, the search for new drugs from macroalgae with pharmaceutical application in the prevention and treatment of hypertension and obesity implies the identification of pure compounds from seaweeds that exhibit such properties. In fact, seaweeds produce a great structural diversity of small molecules with very interesting bioactivities. Here will be presented and discussed the most recent and advanced studies on the pure secondary metabolites from brown, red and green macroalgae that exhibit significant anti-hypertensive and anti-obesity activities. These properties will certainly make them attractive to the pharmaceutical industry as lead compounds in the development of new cardioprotective drugs and, of course, will draw attention to the seaweeds’ health value.

## 2. Anti-Hypertensive Seaweed Compounds

### 2.1. Peptides

**Figure 1:**
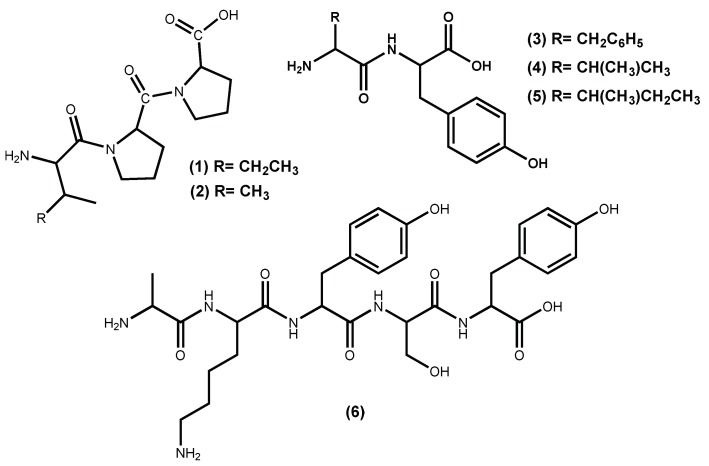
Active peptide structures of the commercial seaweed base-products used to control blood pressure.

Macroalgae protein-derived bioactive peptides possess several beneficial pharmacological properties; among them, the ability to act as antihypertensive agents [29]. Peptides are the most commonly studied natural compounds that inhibit ACE I activity, even the ones isolated from other sources than macroalgae [10,30,31]. Examples of hypotensive commercial peptides generated from macroalgae and with FOSHU (“foods for specified health uses” approved by Japanese Ministry of Health, Labor, and Welfare) antihypertensive claims approved are the Ameal-S 120^®^ (Calpis Co., Ltd., Tokyo, Japan) from Japan and Evolus^®^ (Valio Ltd, Helsinki, Finland) from Finland, which lower blood pressure due to the presence in their formulation of the ACE I inhibitors peptides IPP (**1**) and VPP (**2**) (Figure 1) [32]. Other Japanese products include Wakame Jelly, obtained from the macroalga *Undaria pinnatifida* (Harvey) Suringar (well known as wakame), which contains the peptides FY (**3**), VY (**4**) and IY (**5**) (Figure 1) and Peptide Nori S, obtained from the macroalga *Porphyra yezoensis* (current accepted name accordingly AlgaeBase [33] is *Pyropia yezoensis* (Ueda) M.S.Hwang & H.G.Choi) which contains the peptide AKYSY (**6**) (Figure 1) [29,32,34].

There is evidence that small peptides, ranging in size from 2 to 20 amino acid residues, have revealed promising antihypertensive properties, and this type of peptides can be obtained from seaweed protein hydrolysates [35]. In fact, these peptides with antihypertensive potential are revealed when they are released from the parent protein by digestive enzymes, food processing or by microbial fermentation [29,35]. Moreover, ACE I inhibitory peptides must show resistance to both gastrointestinal proteases and brush border peptidases and be transported intact into the circulatory system to preserve physiological activity [35]. Indeed, Thierney et al. draws attention to the fact that the presence of ACE I inhibitory activity in vitro does not necessarily correlate with an in vivo antihypertensive effect [36].

**Figure 2:**
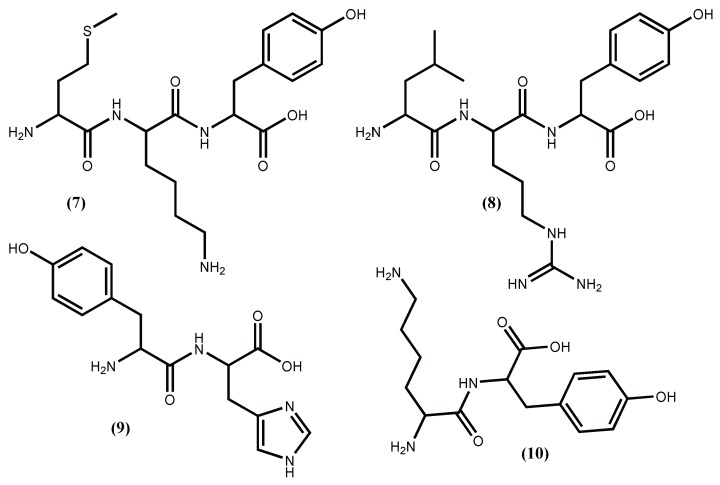
Antihypertensive peptides isolated from *Pyropia yezoensis* (Ueda) M.S.Hwang & H.G.Choi (syn. *Porphyra yezoensis*) and *Undaria pinnatifida* (Harvey) Suringar.

Peptides from *Pyropia yezoensis* (Ueda) M.S.Hwang & H.G.Choi (syn. Porphyra yezoensis), called Nori-peptides, have proven their potent ACE I inhibitory activity in rats [37] and even when tested in humans [38]. The clinical study showed that Nori-peptides (1.8 g/day) induced a significant blood pressure reduction in hypertensive patients without showing a significant change in important clinical parameters [38]. Previous studies allowed the identification of the Nori-peptides amino acid sequences as depicted in Figure 1 for IY (**5**) and AKYSY (**6**) and in Figure 2 for MKY (**7**) and LRY (**8**). Simultaneously, their ACE I inhibitory activity was also evaluated, with IC_50_ values, respectively, of 2.96, 1.52, 7.26 and 5.06 μM [39]. The peptides (**5**), (**7**) and (**8**) showed to be enzyme inhibitors, while peptide (**6**) acted as an enzyme substrate [39]. The peptide (**8**) was synthesized by Furuta et al. [40] and it exhibits ACE I inhibitory activity equivalent to the sesame peptide LVY (IC_50_ 1.80 μM), used as reference in their study, recommended by FOSHU as an antihypertensive agent and included in food and beverages formulations [41]. Saito and Hagino [37] evaluated the antihypertensive effect of these Nori-peptides when administered as a single dose to spontaneously hypertensive rats and confirmed that peptide (**6**) (Figure 1) is, among the Nori-peptides, the most active ACE I inhibitor.

From *Undaria pinnatifida* (Harvey) Suringar, a very popular seaweed-food in the oriental countries that contains 15% protein, Suetsuna et al. [42] isolated and established the amino acid sequences of ten dipeptides with ACE I inhibitory activity. Among them, four dipeptides FY (**3**), IY (**5**) (Figure 1), YH (**9**) and KY (**10**) (Figure 2), exhibit significant in vitro activity, with IC_50_ values of 3.7, 2.7, 5.1 and 7.7 μM, respectively [35,42], while KY (**10**) shows the highest hypotensive effect in vivo (continuous oral administration period assay with 10 mg/day/kg body weight in spontaneously hypertensive rats) perhaps due to greater resistance against protease than the other dipeptides [42].

**Figure 3:**
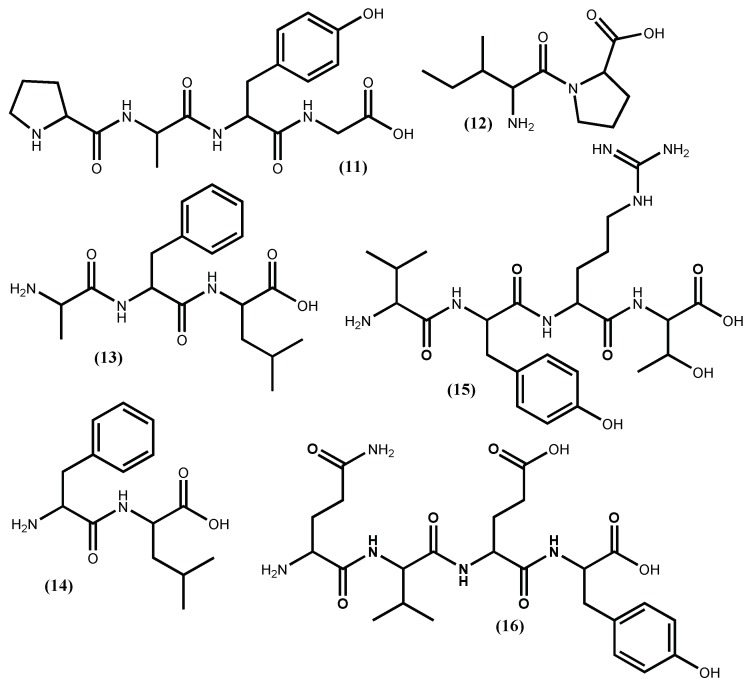
Antihypertensive peptides isolated from *Enteromorpha clathrata*, *Ulva rigida* C.Agardh and *Palmaria palmata* (Linnaeus) F.Weber & D.Mohr.

Recently, it was demonstrated that the tetrapeptide PAFG (**11**) (Figure 3) is a true inhibitor of ACE I and can effectively lower blood pressure, can be orally administered, and has low gastrointestinal enzyme susceptibility [43]. The PAFG (**11**) three hydrophobic amino acid sequence at the *C*-terminal can contribute to its in vitro potent non-competitive ACE I inhibitory activity (IC_50_ 35.9 µM). The authors claimed that PAFG (**11**) was obtained from the hydrolysis of the *Enteromorpha clathrata* protein and that this seaweed is one of the most popular edible marine green seaweeds in Northeast Asian countries, appearing almost year-round [43]. Sadly, the seaweed is not properly identified, and in the AlgaeBase [33], there can be found 58 species with the same name. Furthermore, there are authors suggesting that *Enteromorpha* and *Ulva* are the same genera [44]. The correct source identification is very important, but the use of a positive control is even more important to validate the reported activity, an aspect that is not mentioned by the authors. For our readers, we can suggest the use of captopril, a synthetic clinical drug widely used as antihypertensive, which is very efficient, although it displays some significant side effects [34,45].

Two small peptides, IP (**12**) and AFL (**13**) (Figure 3), were obtained from the *Ulva rigida* C.Agardh protein, through a procedure that involves hydrolysation with pepsin plus bromelain and several purification steps [46]. These peptides revealed ACE I inhibitory activity, the IP (**12**) (IC_50_ 87.6 μM) and AFL (**13**) (IC_50_ 65.9 μM), and peptide (**12**) were shown to be non-competitive while the peptide (**13**) acts as a competitive ACE-inhibitor [46]. The stability assays showed that both peptides are heat-stable and peptide (**13**) is hydrolysed by intestinal mucosa peptidases to a more active dipeptide, the FL (**14**) (Figure 3) (IC_50_ 16.0 μM) acting as a non-competitive ACE I inhibitor, even though it is less active than captopril (IC_50_ 0.77 μM) [46].

The thermolysin hydrolysis of the *Palmaria palmata* (Linnaeus) F.Weber & D.Mohr (well known as dulse) protein originated several ACE I inhibitors peptides, with the most promising one being the LRY (**8**) (Figure 2) followed by VYRT (**15**) (Figure 3) [40]. The absolute quantities of peptides to inhibit 50% of 1.0 U ACE I are, respectively, 0.044 μmol and 0.14 μmol. Although the authors expressed the activity value in a known and acceptable unit with relevant indications of enzyme quantity, its conversion into a more frequent and comparable unit like IC_50_ value in μM is difficult, and because of that it is impossible to promote the peptides potential. The peptide (**15**) was obtained from original *Palmaria palmata* phycobiliprotein, specifically from the α-subunit phycoerythrin, whereas the peptide (**8**) was obtained from the β-subunits phycoerythrin, phycocyanin and allophycocyanin [40].

More recently, a new peptide with ACE inhibitory activity was isolated from the main cultured red macroalga in China with potent economic and ecological value *Gracilariopsis lemaneiformis* (Bory de Saint-Vincent) E.Y.Dawson, Acleto & Foldvik, after algal protein hydrolysis with trypsin [47]. The new peptide was identified as QVEY (**16**) (Figure 3) and showed an IC_50_ value of 474.36 μM [39], an uninteresting level of activity in view of its application as drug.

Five different enzymatic digests of the brown seaweed *Ecklonia cava* Kjellman aqueous extract, obtained at 70 °C, exhibited potent ACE I inhibitory effects with IC_50_ values from 2.33 up to 3.56 μg/mL [48]. Also, the enzymatic hydrolysis of *Pyropia columbina* (Montagne) W.A.Nelson proteins produced two fractions that exhibit higher ACE I inhibition activity (IC_50_ 1.2 ± 0.1 mg/mL) than the crude protein, and presented an uncompetitive mechanism of action [49]. Although these are interesting results, the authors did not purify the active peptides and consequently their amino acid sequence is unknown. The structures of the active peptides and/or their progenitor protein are of most importance to establish relationships between structures and inhibition mechanisms. The structure-activity relationship of marine-derived ACE I inhibitor peptides is far away from being established, but some characteristics that might help can be highlighted. It seems that ACE I potent inhibitors have: i) hydrophobic amino acid residues in the *C*-terminal first three positions; ii) tryptophan, phenylalanine, tyrosine, or proline at their *C*-terminal, and branched aliphatic amino acid at the *N*-terminal; iii) a positively charged residue at *C*-terminal adjacent to an aromatic residue; iv) proline residue, especially at the *C*-terminal, which contributes to low peptides degradation by digestive enzymes [42,50,51,52].

From the above-mentioned results, it can be inferred that regular ingestion of seaweeds could be effective to maintain blood pressure at a healthy level, due to their peptides potent ACE-inhibitory activity. From the peptides reported until today, it is obvious that the dipeptide (**5**) and the pentapeptide (**6**) (Figure 1) are the most potent ACE I inhibitors isolated from seaweeds.

**Figure 4:**
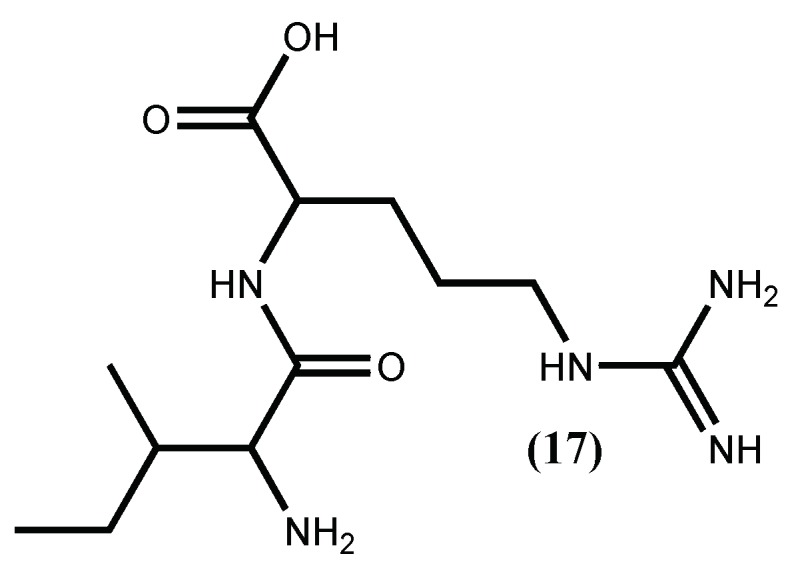
Renin-inhibitor peptide isolated from *Palmaria palmata* (Linnaeus) F.Weber & D.Mohr.

The inhibition of ACE I enzyme is the most common target applied in hypertension therapy, largely due to the success of the synthetic ACE I inhibitor captopril. However, the treatment with captopril and other ACE I inhibitors entails secondary effects, such as dry cough or angioneurotic oedema. Therefore, inhibition of renin, the initial rate-limiting enzyme in the RAS, has advantages over ACE I inhibition. It is a very specific enzyme (the only known enzyme that converts angiotensinogen to angiotensin I), where the vasodilator bradykinin is not involved, so the above-mentioned secondary effects are not expected [45,53]. For this reason, the interest in finding new renin inhibitors has increased, and macroalgae peptides seem to be a good choice [54,55]. The first renin inhibitory activities for peptides isolated from a macroalgae was, in fact, done with the protein from red seaweed *Palmaria palmata* (Linnaeus) F.Weber & D.Mohr. This protein was hydrolysed, with papain, producing the tridecapeptide, for which the sequence was established as IRLIIVLMPILMA and its renin inhibitory activity was low (IC_50_ 3.34 mM) [54]. Later on, the same group demonstrated its ability to impart an antihypertensive effect in vivo after oral administration of 50 mg/kg body weight of the tridecapeptide and additionally, using a combination of in silico cleavage analysis coupled with in vitro simulated gastrointestinal digestion, that this tridecapeptide is cleaved through the gastric digestion and activated to the dipeptide IR (**17**) (Figure 4) [55]. Previous work [56] showed that this dipeptide, previously isolated from a pea peptide hydrolysate, inhibited renin and ACE I activities at concentrations of 3.5 mM.

The kinin−nitric oxide system works in concert with the RAS system to regulate blood pressure. The intermediate involved in the kinin–nitric oxide system, bradykinin, activates Ca^2+^/calmodulin-dependent endothelial nitric oxide synthase (eNOS), which catalyzes the conversion of arginine to nitric oxide (NO), leading to a drop in blood pressure. There is evidence that eNOS knockout and NO deficiency can lead to clinical hypertension [57]. Therefore, arginine-rich peptides can also be considered as a strategy for hypertension therapy once they act as a source of nitric oxide, which in turn plays important physiological roles in the vascular endothelium and thus can produce in vivo vasodilator effects during hypertension [58]. The effect of arginine-rich peptides as antihypertensive was recently reviewed [59] and the majority of the arginine-rich peptides were obtained from foodstuffs. However, some macroalgae species like *Porphyra* spp., *Chondrus crispus* spp. and *Ulva pertusa* Kjellman contain high levels of arginine [60]; thus, at least these species should be seen as potential sources of antihypertensive arginine-rich peptides.

Taking into account the great application of peptides ACE I inhibitors in promoting cardiovascular health, the extraction procedures optimization gathered several attention and discussion about criteria for selecting extraction methods and the extracts quality [22,35,61]. For example, recently, the extraction with cellulase and α-amylase hydrolysis was compared with the conventional method of maceration. The two procedures were tested in several brown seaweeds and it was evident that the maceration procedure extracts were less rich in compounds with ACE I inhibitory activity [62]. However, the authors proposed that the ACE I inhibitory activity of the extracts obtained by enzymatic extraction may be due to the presence of phlorotannins and carbohydrates, and not to peptides [62].

### 2.2. Phlorotannins

Phloroglucinol polymerization gives a family of important natural compounds, the phlorotannins, which are highly hydrophilic and have a wide range of molecular sizes, ranging between 126 Da and 650 kDa. Their occurrence in brown seaweeds is very common, mainly in *Ecklonia* species, and their various beneficial biological activities, such as anticancer, antidiabetic, antiallergic, antioxidant and antihypertensive activities [63,64,65,66] are also recognized.

**Figure 5:**
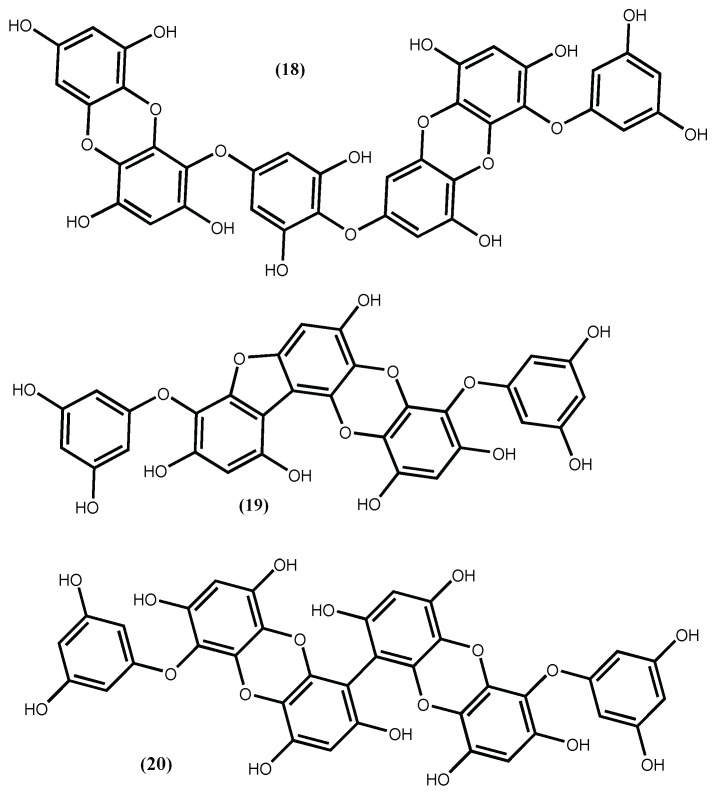
Antihypertensive phlorotannins isolated from *Ecklonia cava* Kjellman and *Ecklonia stolonifera* Okamura.

The involvement of phlorotannins in the ACE I inhibitory activity proposed by Olivares-Molina and Fernández [62] is not odd, because it was previously detected. For instance, from ethanolic extract of *Ecklonia cava* Kjellman were isolated phlorotannins that exhibited potential ACE inhibition activity and from which the most active was dieckol (**18**) (Figure 5), with an ACE inhibitor IC_50_ 1.47 ± 0.04 mM [67]. Dieckol (**18**) was found to be a non-competitive inhibitor against ACE I, with an inducible effect on the production of NO in EAhy926 cells and without having cytotoxic effects, although its inhibitory capacity is not comparable with the one presented by captopril (IC_50_ 0.025 ± 0.90 μM) [67]. We draw the reader’s attention to the fact that captopril is a clinical drug widely used as an antihypertensive. The low IC_50_ reported seems to be in accordance with that; however, the associated standard deviation is several times higher than the IC_50_ mean value, which is unacceptable. In our opinion, this could be a typographical error.

The compound dieckol (**18**) was also isolated from the edible brown alga *Ecklonia stolonifera* Okamura along with phlorofucofuroeckol A (**19**) (Figure 5), with the compound **19** being an ACE I inhibitor much stronger (with an IC_50_ value of 12.74 ± 0.15 μM) than the compound **18,** although not so active as captopril [68].

The 6,6′-bieckol (**20**) (Figure 5) isolated from *Ecklonia cava* Kjellman, inhibits the ACE enzyme with an IC_50_ value of 0.42 mM [69], being less active than phlorofucofuroeckol A (**19**) (Figure 5). Additionally, Ko et al. [69], using docking studies, determined that 6,6′-bieckol (**20**) (Figure 5), might interact with the S1, S′1 and S′2 pockets of ACE and then restrain the ACE activity; using human umbilical vein endothelial cells (HUVECs) assay, they demonstrate that 6,6′-bieckol generates endothelial nitric oxide (eNOS)-mediated nitric oxide (NO) by activating Akt; and using spontaneously hypertensive rat models, 6,6′-bieckol (**20**) (Figure 5) causes great reduction of systolic blood pressure at a dose of 20 mg/kg body weight and injected orally. These results are good indicators that 6,6′-bieckol (**20**) has potential to be used in the treatment of hypertension.

The phlorotannins structure-activity relationships are incomplete, but it seems that a dibenzo-1,4-dioxin moiety may be crucial to promote ACE I inhibition. Moreover, an additional dibenzofuran ring may also increase the inhibitory effect [68]. There is evidence that phlorotannins ACE I inhibitory activity is due to their protein-binding ability and the consequent decrease efficiency of ACE I after binding [67]. This protein-binding ability depends on the length and structure of the phlorotannin, apparently phloroglucinol pentamers or hexamers are better inhibitors [70].

### 2.3. Polysacharides

Endothelin1 (ET 1), the predominant compound of the endothelin system, acts through intracellular pathways of two endothelin receptors (ETA and ETB). Endothelial cells regulate vascular tone and provoke mitogenic and pro-inflammatory reactions. There is evidence that the blockade of endothelin receptors, particularly the ETA subtype, can be a strategy to treat the major cardiovascular pathologies [71,72,73].

**Figure 6:**
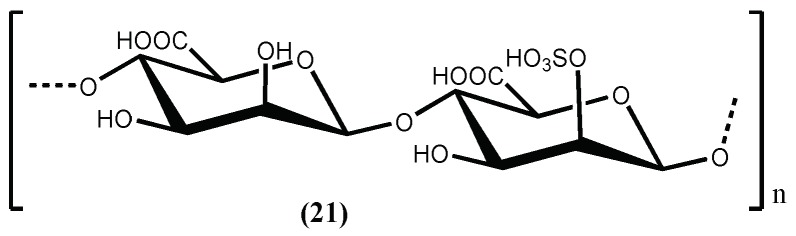
The antihypertensive D-polymannuronic sulfate isolated from *Pelvetia canaliculata* (Linnaeus) Decaisne & Thuret.

D-Polymannuronic sulphate (**21**) (Figure 6), a carbohydrate type compound, can be obtained from the brown alga *Pelvetia canaliculata* (Linnaeus) Decaisne & Thuret, and also demonstrate in vivo acute and prophylactic hypotensive potency. D-Polymannuronic sulphate (**21**) displayed therapeutic potency (50 mg/kg) comparable to that of captopril (14 mg/kg). The results indicate that D-polymannuronic sulphate (**21**) promotes the elevation of NO contents and lowered the concentrations of Ang II and ET 1 [74], mainly by dose-dependently reducing and/or preventing the increase of systolic blood pressure and by decreasing the heart rate with the reduction of arterial blood pressure [74].

As a summary, the effects of secondary metabolites as anti-hypertensive agents and the level of activity are shown in Table 1.

The above discussed results indicate that the macroalgae referred to can and should be considered as sources of hypotensive agents, for which the application in medicine is of particular relevance to control hypertension. However, Table 1 shows, very clearly, how little is known about their mechanism of action and points out an area whose scientific research must be deepened.

## 3. Anti-Obesity Seaweed Compounds

Obesity is a medical condition in which excess body fat has accumulated to the extent that it may have an adverse effect on many diseases including diabetes, hypertension and other cardiovascular complications [75,76]. There is evidence that seaweed ingestion can control obesity, for instance a mixture of brown seaweed and pomegranate seed, called xanthigen showed anti-obesity activity through inhibition of peroxisome proliferator-activated receptor γ (PPARγ) expression and activation of the AMP-activated protein kinase (AMPK) phosphorylation [77]. There is also evidence that their chemical components can become potential drugs to be used in obesity treatment [27,78,79]. Although some in vivo studies report the anti-obesity of several seaweeds, the whole alga [80,81] or ethanolic extracts [82] were used in the evaluations, their chemical constituent evaluations is less reported, and use in in vivo models is scarce.

Recent in vivo studies revealed that enzymatic-digested alginate oligomers, a polysaccharide fraction isolated from brown seaweeds, can induce anti-obesity effects [83,84]; however, the chemical characterization of this alginate fraction is not reported. On the other hand, some interesting in vitro studies can be found in the literature and can be regarded as a starting point for further research trying to identify the active principle and its mechanism of action.

### 3.1. Phlorotannins

**Figure 7:**
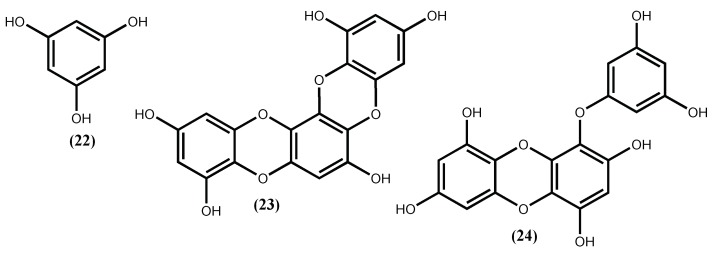
Some anti-adipogenic compounds isolated from the edible brown alga *Ecklonia stolonifera* Okamura.

One of the anti-obesity strategies targets the inhibition of adipocyte differentiation [85,86,87]. For example, Jung group’s [88] dedicated part of their work studying the bioactive compounds isolated from the edible brown alga *Ecklonia stolonifera* Okamura and found out that five phlorotannins with low and high molecular weight (MW from 126 to 742) reduced lipid accumulation in 3T3-L1 cells in a dose-dependent manner (12.5–100 µM) being the phlorofucofuroeckol A (**19**) (Figure 5) the most active one (IC_50_ 17.86 μM). Apparently, the activity depends on the molecular weight, the lower molecular weight phlorotannins phloroglucinol (**22**), dioxinodehydroeckol (**23**), and eckol (**24**) (Figure 7), with MW from 126 to 372, exhibit potent inhibitory activities on adipocyte differentiation, whereas the highest molecular weight dieckol (**18**) (Figure 5) only exerted weak anti-adipogenesis activity. The results indicate that these compounds suppress C/EBPα and PPARγ expression and this action may explain the *Ecklonia stolonifera* Okamura effects on obesity [88].

Phlorotannin 6,6′-bieckol (**20**) (Figure 5), was isolated from a brown seaweed customarily in Korean cuisine, the *Ecklonia bicyclis* Kjellman (syn. *Eisenia bicyclis* (Kjellman) Setchell [89]. The biological assay demonstrates that phlorotannin (**20**) strongly suppressed lipid accumulation in 3T3-L1 adipocytes in a dose-dependent manner. Moreover, at a concentration of 67.3 μM did not provoke cytotoxic effects. According to the authors, its action mechanism involves the inhibition of lipogenic enzymes and also the inhibition of several transcription factors mRNA expression [89].

### 3.2. Sterols

**Figure 8:**
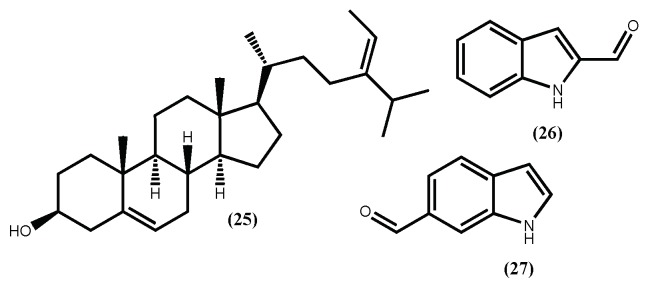
Some compounds isolated from brown seaweeds that inhibit the cells adipocyte differentiation.

As a part of these authors continuous search for anti-obesity agents, fucosterol (**25**) (Figure 8) was evaluated for its potential to inhibit adipocyte differentiation and lipid formation [90]. Fucosterol (**25**) decrease the expression of the adipocyte marker proteins PPARγ and CCAAT/enhancer-binding protein (C/EBPα) in a concentration-dependent manner (3.125–50 μM). Moreover, at concentrations up to 50 μM, fucosterol (**25**) did not present cytotoxicity. In addition to the mechanism described above, Lee et al. [91], showed that fucosterol (**25**) isolated from *Ecklonia stolonifera* Okamura, exhibits the ability to inhibit adipogenesis of 3T3-L1 preadipocytes through downregulation of SREBP1 and modulation of multiple signaling pathways including PI3K/Akt and ERK-dependent FoxO signaling pathway. This fucosterol (**25**) activity contributes to emphasizing the *Ecklonia stolonifera* Okamura potential as a source of anti-obesity compounds.

### 3.3. Indole Derivatives

Recently, from another brown seaweed, the *Sargassum thunbergii* (Mertens ex Roth) Kuntze, several indole derivatives were isolated and their adipogenesis inhibition was evaluated [92]. From the several indoles, two can be highlighted, the 1*H*-indole-2-carbaldehyde (**26**) and 1*H*-indole-6-carbaldehyde (**27**) (Figure 8), due to their non-toxic and effective inhibition of the 3T3-L1 cells adipocyte differentiation. The authors also demonstrate that these indoles’ inhibition mechanism is through the activation of the AMPK signal pathway. These findings not only establish the *Sargassum thunbergii* (Mertens ex Roth) Kuntze anti-obesity effect but also suggest that indoles (**26**) and (**27**) can prevent obesity [92].

### 3.4. Caretonoids

**Figure 9:**
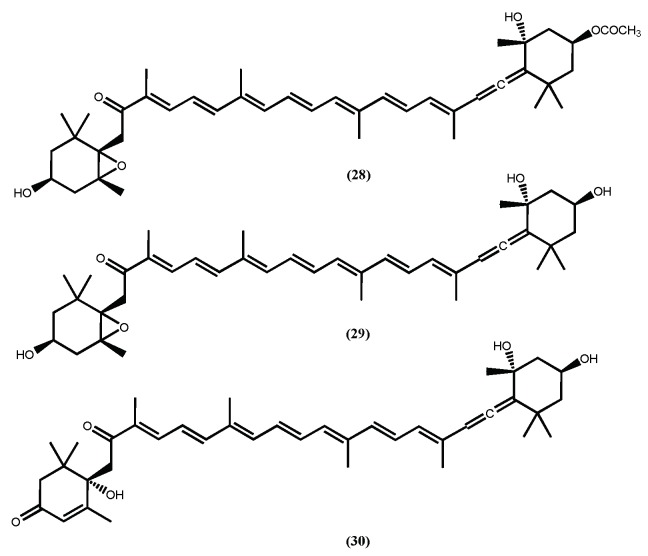
The structures of fucoxanthin (**28**) fucoxanthinol (**29**) and amarouciaxanthin A (**30**) with anti-obesity effects.

Fucoxanthin (**28**) (Figure 9) is probably the most recognized secondary metabolite found in macroalgae, and its biological properties are well established [93]. Among all activities, the anti-obesity is almost certainly the most studied one [94,95], in fact its anti-obesity effects were recently reviewed [22,95,96] and the authors stress the efficacy, detailed description of the action mechanism and safety of this pharmaceutical ingredient in in vivo assays. Herein we highlight a few interesting results, such as the ability of fucoxanthin (**28**) to inhibit the intercellular lipid accumulation by reducing the expressions of PPAR𝛾, C/EBP𝛼, and SREBP1c during the intermediate and late stages of differentiation [97] and its ability to stimulate uncoupling protein-1 (UCP-1) and β3-adrenergic receptor expression in white adipose tissue (WAT) and thus augment lipolysis and thermogenesis [95,96], contributing to significantly attenuated weight gain. Additionally, fucoxanthinol (**29**) and amarouciaxanthin A (**30**) (Figure 9), fucoxanthin (**28**) metabolites, also showed the ability to downregulate PPAR𝛾 and even exhibited stronger suppressive effects than fucoxanthin (**28**) on adipocyte differentiation in 3T3-L1 cells [98,99]. It is curious that the more recent studies involving fucoxanthin (**28**) are more dedicated to finding efficient systems for its delivery and naturally to establish that the system does not reduce the fucoxanthin (**28**) anti-obesity activity [100] or exhibit toxicity [101].

As a summary, the mechanisms involved in the secondary metabolites’ anti-obesity effect and how they affect the targets are shown in Table 2.

## 4. Conclusions

Based on the above examples, it is obvious that pure secondary metabolites from seaweeds represent promising anti-hypertensive and anti-obesity agents with considerably high activities (IC_50_ values in the micromolar range), through the counteracting of key mechanisms underlying the onset of such disorders. The most active ACE inhibitors reported are the peptides IY (**5**) and AKYSY (**6**) (IC_50_ 1.52–2.96 μM) while the best phlorotannin with ACE I inhibitor activity reported is phlorofucofuroeckol A (**19**) (IC_50_ 12.74 μM). Compounds that act in other antihypertensive targets, such as RAS system or nitric oxide synthase activators, were also reported however, in our opinion, their real pharmacological potential needs to be confirmed.

Fucoxanthin (**28**) seems to be the most studied and promising anti-obesity compound, while the 6,6′-bieckol (**20**) seems to be the most interesting phlorotannin since it is able to strongly suppress the lipid accumulation in 3T3-L1 adipocytes.

The careful analysis of the results most frequently reported shows that the assays performed with pure seaweed compounds are mainly in vitro. Only a few in vivo studies were published, which is indicative that more research using in in vivo models is required. Furthermore, it is also clear that extra efforts are necessary to: i) fully understand the structure-activity relationships and the cellular mechanisms of action; ii) prove the non-toxicity of the most promise compounds, before the seaweeds secondary metabolites are approved for medicinal applications.

On the other hand, the studies discussed in points 2 and 3 do not address the question of the most appropriate form of intake, whether in the form of a drug, nutraceutical or dietary supplement. However, given that many of the algae in which antihypertensive and anti-obesity compounds have already been identified are edible algae, it will be anticipated that, at an early stage, the benefits of these compounds will be from functional foods and only later as drugs.

We hope that this revision will provide inspiration for such detailed research, which can result in preclinical and clinical trials of specific seaweed compounds and boost their value as a resource of potential antihypertensive and anti-obesity useful drugs.

## Figures and Tables

**Table 1:** Anti-hypertensive effect of the pure secondary metabolites isolated from seaweeds.

Secondary metabolite (N^o^)	Effects ^1^ and mechanism ^2^ IC_50_^3^	Reference
*ACE I inhibitors*		
Peptide IPP (**1**)	Positive inhibition; ↓ Blood pressure	[32]
Peptide VPP (**2**)	Positive inhibition; ↓ Blood pressure	[32]
Peptide FY (**3**)	Positive inhibition; ↓ Blood pressure	[33]
Peptide VY (**4**)	Positive inhibition; ↓ Blood pressure	[33]
Peptide IY (**5**)	IC_50_ 2.96 µM; ↓ Blood pressure	[33,39]
Peptide AKYSY (**6**)	IC_50_ 1.52 µM; ↓ Blood pressure	[29,32,34,39]
Peptide MKY (**7**)	IC_50_ 7.26 µM; ↓ Blood pressure	[39]
Peptide LRY (**8**)	IC_50_ 5.06 µM; ↓ Blood pressure	[39,40]
Peptide YH (**9**)	IC_50_ 5.1 µM; ↓ Blood pressure	[35,42]
Peptide KY (**10**)	IC_50_ 7.7 µM; ↓ Blood pressure	[35,42]
Peptide PAFG (**11**)	IC_50_ 35.9 µM; ↓ Blood pressure	[43]
Peptide IP (**12**)	IC_50_ 87.6 µM	[46]
Peptide AFL (**13**)	IC_50_ 65.9 µM	[46]
Peptide PAFG (**14**)	IC_50_ 16.0 µM	[46]
Peptide VYRT (**15**)	Positive inhibition	[40]
Peptide QVEY (**16**)	IC_50_ 474.36 µM	[47]
Dieckol (**18**)	IC_50_ 1470 µM; ↑ production of NO in EAhy926 cells	[67]
Phlorofucofuroeckol A (**19**)	IC_50_ 12.74 µM;	[68]
6,6′-Bieckol (**20**)	IC_50_ 0.42 mM; interact with the S1, S′1 and S′2 pockets of ACE; ↑ eNOS-mediated NO in HUVEC cells; ↓ Systolic blood pressure	[69]
D-Polymannuronic sulphate (**21**)	Positive inhibition; ↑ production of NO; ↓ concentrations of Ang II; ↓ concentrations of ET 1; ↓Blood pressure	[74]
*RAS inhibitors*		
Peptide QVEY (**17**)	Positive inhibition	[55]

ACE I = angiotensin-I converting enzyme; Ang II = angiotensin II; ET 1 = endothelin1; RAS = renin-angiotensin system; eNOS-mediated NO = endothelial nitric oxide-mediated nitric oxide; HUVEC = human umbilical vein endothelial cells; ^1^ Only when the effect was actually detected; ^2^ When data are available; ^3^ Only the IC_50_ values in µM are considered.

**Table 2:** Anti-obesity effect of the pure secondary metabolites isolated from seaweeds.

Secondary metabolite (N^o^)	Target and activity	Reference
Dieckol (**18**)	Reduced lipid accumulation in 3T3-L1cells; ↓ C/EBPα and PPARγ expression	[88]
Phlorofucofuroeckol A (**19**)	Reduced lipid accumulation in 3T3-L1 cells (IC_50_ 17.86 μM); ↓ C/EBPα and PPARγ expression	[88]
6,6′-Bieckol (**20**)	Suppressed lipid accumulation in 3T3-L1 adipocytes; inhibition of lipogenic enzymes; ↓ mRNA expression	[89]
Phloroglucinol (**22**)	Reduced lipid accumulation in 3T3-L1 cells; potent inhibitory activities on adipocyte differentiation; ↓ C/EBPα and PPARγ expression	[88]
Dioxinodehydroeckol (**23**)	Reduced lipid accumulation in 3T3-L1; potent inhibitory activities on adipocyte differentiation; ↓ C/EBPα and PPARγ expression	[88]
Eckol (**24**)	Reduced lipid accumulation in 3T3-L1 cells; potent inhibitory activities on adipocyte differentiation; ↓ C/EBPα and PPARγ expression	[88]
Fucosterol (**25**)	↓ C/EBPα and PPARγ expression; inhibited adipogenesis of 3T3-L1; ↓ SREBP; modulation of PI3K/Akt- and ERK-dependent FoxO signalling pathways	[90,91]
1*H*-Indole-2-carbaldehyde (**26**)	inhibition of the 3T3-L1 cells adipocyte differentiation; ↑AMPK signal pathway	[92]
1*H*-Indole-6-carbaldehyde (**27**)	inhibition of the 3T3-L1 cells adipocyte differentiation; ↑AMPK signal pathway	[92]
Fucoxanthin (**28**)	inhibit the intercellular lipid accumulation; ↓ C/EBPα and PPARγ expression; ↓ SREBP; ↑ uncoupling protein-1 (UCP-1); ↑ β3-adrenergic receptor expression	[95,96,97]
Fucoxanthinol (**29**)	↓ PPARγ expression; ↓ adipocyte differentiation in 3T3-L1 cells	[98,99]
Amarouciaxanthin A (**30**)	↓ PPARγ expression; ↓ adipocyte differentiation in 3T3-L1 cells	[98,99]

3T3-L1 = cell line derived from (mouse) 3T3 cells; C/EBPα = C/enhancer binding protein alpha; PPARγ = Peroxisome proliferator-activated receptor gamma; mRNA = messenger ribonucleic acid; SREBP = Sterol regulatory element-binding proteins; PI3K/Akt = phosphoinositide 3-kinase (also known as Akt; ERK = extracellular signal-regulated kinase; FoxO = Forkhead box O; AMPK = 5' adenosine monophosphate-activated protein kinase; UCP-1 = Mitochondrial uncoupling proteins 1.
